# Supplementary material for: Transcriptome Analysis of Sogatella furcifera (Homoptera: Delphacidae) in Response to Sulfoxaflor and Functional Verification of Resistance-Related P450 Genes
Source: Int J Mol Sci. 2019 Sep 15;20(18):4573. doi: 10.3390/ijms20184573 (PMC6770238; doi:10.3390/ijms20184573)
Supplement: Supplementary file 1 [file ijms-20-04573-s001.pdf]

Supplementary Table 1. Statistics of transcriptome data filtering in the six samples

Sample	Before Filter Reads Num	After Filter clean Reads Num (%)	GC	Adapter (%)	Low quality (%)
Sus-Lab1	50653904(Q20:97.98%, Q30:94.92%)	49806332(Q20:98.72%, Q30:96.12%)	45.71%	203180 (0.4%)	641366 (1.27%)
Sus-Lab2	55748732(Q20:97.91%, Q30:94.77%)	54762862(Q20:98.69%, Q30:96.04%)	46.42%	232514 (0.42%)	750092 (1.35%)
Sus-Lab3	59092434(Q20:98.04%, Q30:95.07%)	58128812(Q20:98.76%, Q30:96.23%)	45.36%	231182 (0.39%)	729124 (1.23%)
SF-Sel1	53682042(Q20:97.95%, Q30:94.86%)	52776552(Q20:98.7%, Q30:96.07%)	45.55%	200350 (0.37%)	701990 (1.31%)
SF-Sel2	56892728(Q20:97.93%, Q30:94.84%)	55908308(Q20:98.7%, Q30:96.1%)	45.53%	221788 (0.39%)	759308 (1.33%)
SF-Sel3	59049302(Q20:97.98%, Q30:94.97%)	58066616(Q20:98.73%, Q30:96.19%)	43.90%	212374 (0.36%)	766902 (1.3%)

Note: Reads Num (PE) = Reads1 Num + Reads2 Num (the reverse and forward total reads); the probability of error recognition of Q20 and Q30 were 1% and 0.1%, respectively.

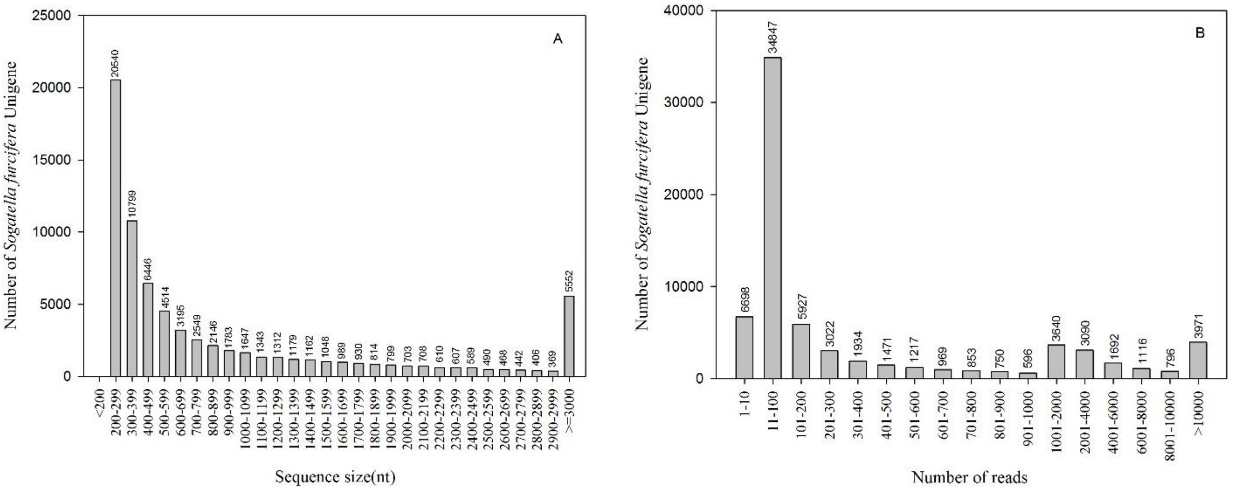

Supplementary figure 1. Length distribution of unigene in the transcriptome data *of S. furcifera* reads and coverage map of reads in unigene. (A) Distribution of unigenes in different length ranges (e.g.,<200 represents 0–200). (B) Distribution of unigenes with different number of reads (e.g.,1-10 represents unigene consisted with 1-10 reads).

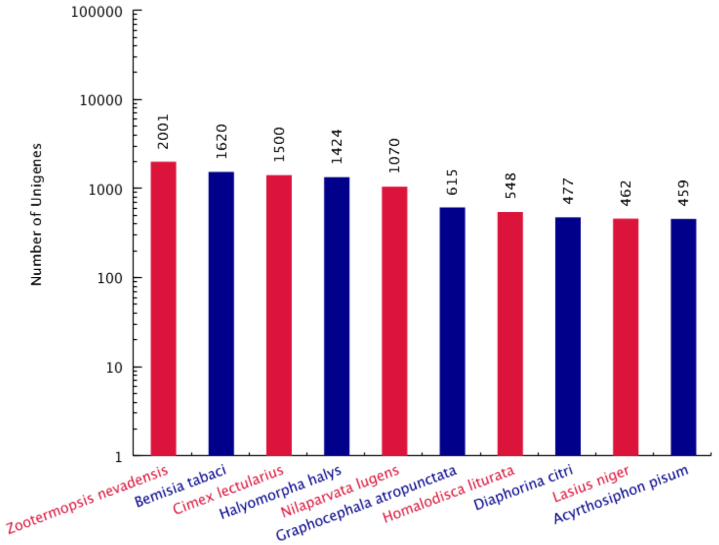

Supplementary Figure 2A. Species distribution of the BlastX matchs to the unigene on the Nr database (Top ten)

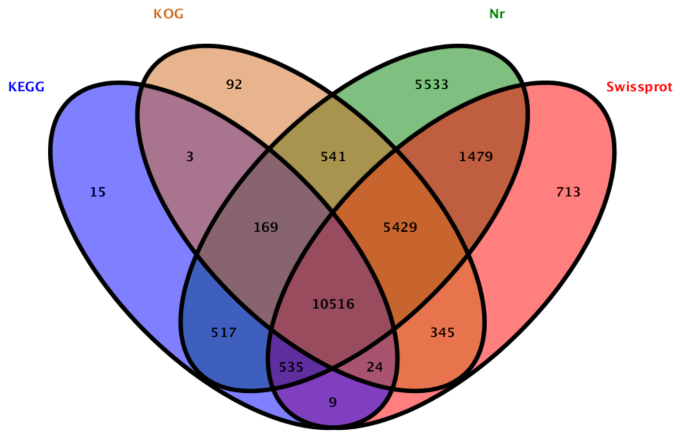

Supplementary Figure 2B. Venn Diagram of annotation genes of four database

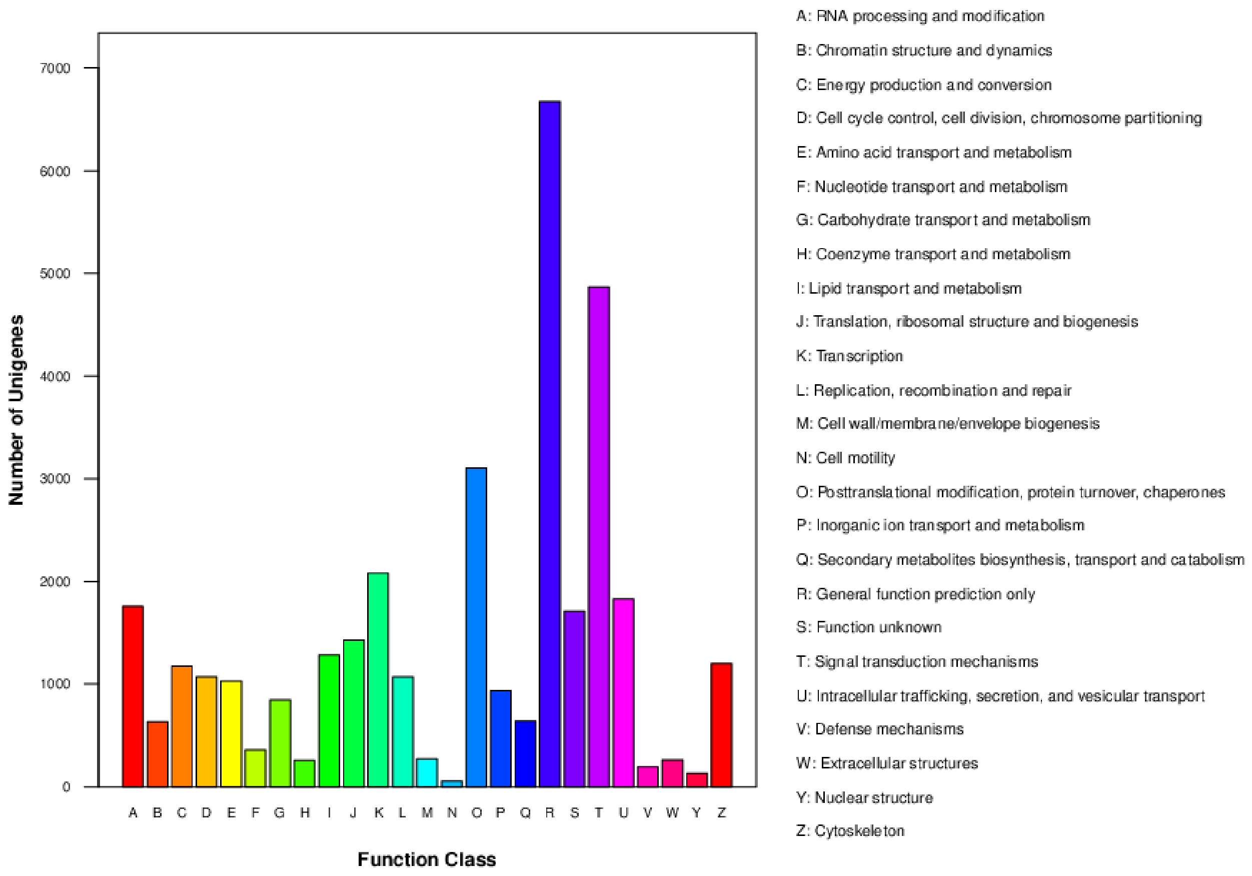

Supplementary Figure 2C. KOG function classification of *Sogatella_furcifera.* All putative proteins were aligned to the KOG database and were classified functionally into at least 25 molecular families.

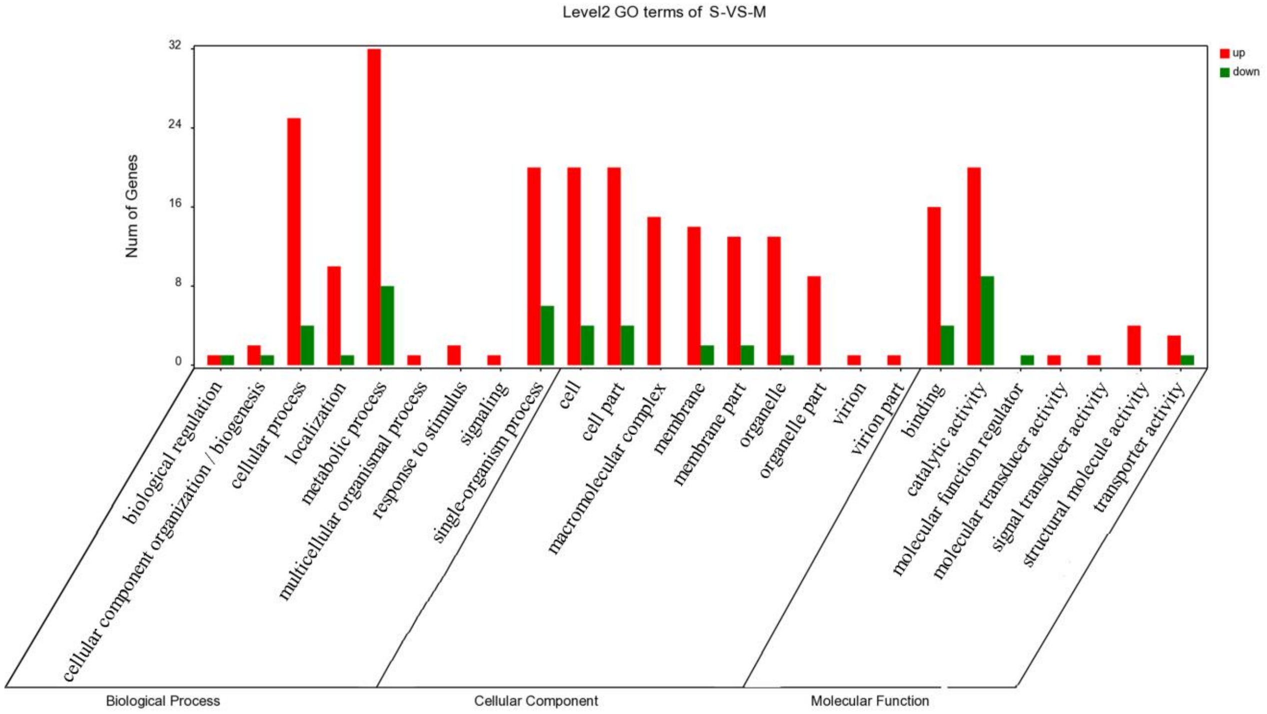

Supplementary Figure 3. GO categories of unigene on the different groups. The ordinate means GO terms, the abscissa means the number of DEGs of each GO term. Red color means the terms of up-regulated genes, green color means the terms of down regulated genes, respectively.

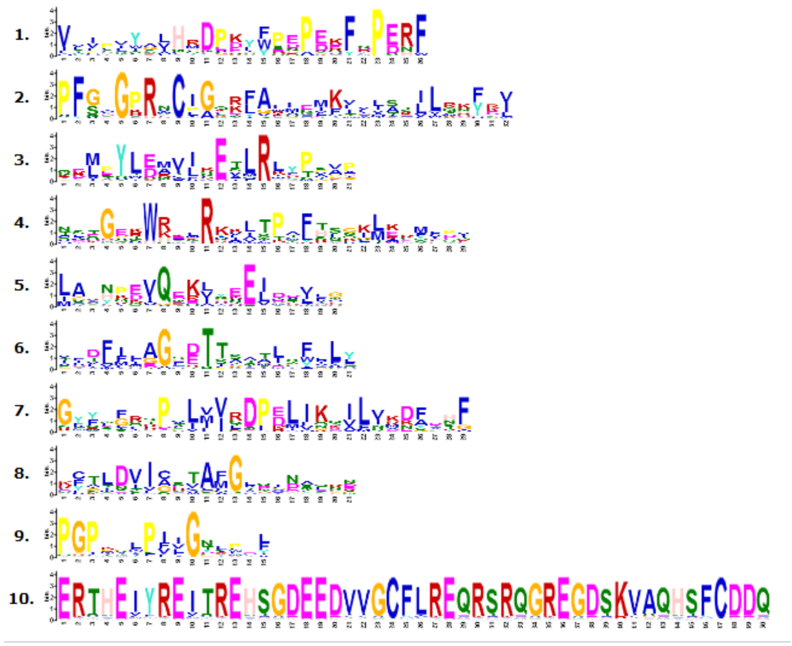

Supplementary Figures 4 Conservative sequence display of different motif structures. The number of 1 to 10 stands for motif1 to motif10, and abscissa represents conserved amino acid sequences of different motifs.
